# The Vitamin Nicotinamide: Translating Nutrition into Clinical Care

**DOI:** 10.3390/molecules14093446

**Published:** 2009-09-09

**Authors:** Kenneth Maiese, Zhao Zhong Chong, Jinling Hou, Yan Chen Shang

**Affiliations:** 1 Division of Cellular and Molecular Cerebral Ischemia, Wayne State University School of Medicine, Detroit, Michigan 48201, USA; 2 Departments of Neurology and Anatomy & Cell Biology, Wayne State University School of Medicine, Detroit, Michigan 48201, USA; 3 Barbara Ann Karmanos Cancer Institute, Wayne State University School of Medicine, Detroit, Michigan 48201, USA; 4 Center for Molecular Medicine and Genetics, Wayne State University School of Medicine, Detroit, Michigan 48201, USA; 5 Institute of Environmental Health Sciences, Wayne State University School of Medicine, Detroit, Michigan 48201, USA

**Keywords:** Alzheimer’s disease, diabetes, erythropoietin, forkhead transcription factors, Wnt

## Abstract

Nicotinamide, the amide form of vitamin B_3 _(niacin), is changed to its mononucleotide compound with the enzyme nicotinic acide/nicotinamide adenylyl-transferase, and participates in the cellular energy metabolism that directly impacts normal physiology. However, nicotinamide also influences oxidative stress and modulates multiple pathways tied to both cellular survival and death. During disorders that include immune system dysfunction, diabetes, and aging-related diseases, nicotinamide is a robust cytoprotectant that blocks cellular inflammatory cell activation, early apoptotic phosphatidylserine exposure, and late nuclear DNA degradation. Nicotinamide relies upon unique cellular pathways that involve forkhead transcription factors, sirtuins, protein kinase B (Akt), Bad, caspases, and poly (ADP-ribose) polymerase that may offer a fine line with determining cellular longevity, cell survival, and unwanted cancer progression. If one is cognizant of the these considerations, it becomes evident that nicotinamide holds great potential for multiple disease entities, but the development of new therapeutic strategies rests heavily upon the elucidation of the novel cellular pathways that nicotinamide closely governs.

## 1. Introduction

**Figure 1:**
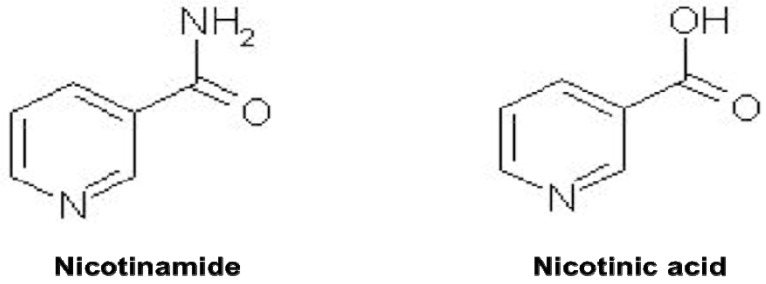
Chemical structures of nicotinamide and nicotinic acid.

Nicotinamide (Figure 1) is the amide form of vitamin B_3 _(niacin) and is obtained through synthesis in the body or as a dietary source and supplement [1]. Nicotinic acid is the other form of the water-soluble vitamin B_3 _(Figure 1). Although also present from animal sources, the principal form of niacin in dietary plant sources is nicotinic acid that is rapidly absorbed through the gastrointestinal epithelium [2]. Nicotinamide is subsequently generated through the conversion of nicotinic acid in the liver or through the hydrolysis of NAD^+^. Once nicotinamide is obtained in the body, it functions as the precursor for the coenzyme ß-nicotinamide adenine dinucleotide (NAD^+^) [3,4] and also is essential for the synthesis of nicotinamide adenine dinucleotide phosphate (NADP^+^) [5]. Initially, nicotinamide is changed to its mononucleotide form (NMN) with the enzyme nicotinic acid/nicotinamide adenylyltransferase yielding the dinucleotides NAAD^+^ and NAD^+^. NAAD^+^ also yields NAD^+^ through NAD^+^ synthase [6] or NAD^+ ^can be synthesized through nicotinamide riboside kinase that phosphorylates nicotinamide riboside to NMN [7,8]. These cellular pathways are essential for energy metabolism and may directly impact normal physiology, as well as disease progression [9,10,11,12].

In deficiency states, lack of nicotinamide can lead to fatigue, loss of appetite, pigmented rashes of the skin, and oral ulcerations. More severe states of deficiency lead to pellagra that is characterized by cutaneous rashes, oral ulcerations, gastrointestinal difficulties, and cognitive loss. Pellagra can occur during low nicotinamide conditions or due to the inability to absorb nicotinamide. For example, inability to absorb tryptophan that causes Hartnup’s disease, isoniazid treatment, or carcinoid syndrome also can be associated with pellagra. Excessive alcohol consumption that is associated with poor dietary intake also can lead to severe nicotinamide loss and insufficient gastrointestinal absorption.

## 2. Nicotinamide, Oxidative Stress, and Cellular Survival

Ultimate cellular survival can be determined by a number of factors, but the process of apoptosis can represent one of the critical pathways for a number of disease entities. Apoptosis can contribute to disorders such as diabetes [13,14,15,16], tissue ischemia [17,18,19,20], bone fatigue [21], Alzheimer's disease [22,23,24,25,26,27,28,29,30,31,32], neurodegenerative disorders [33,34,35,36], plasticity associated with ischemic preconditioning [37], aging-related diseases [38,39,40], and toxic conditions during development [41,42]. The pathology with these disorders can be linked to mitochondrial dysfunction [43,44,45,46], especially during metabolic disorders [47] and Alzheimer’s disease [48], that ultimately can lead to cell death in a variety of cells such as neurons, endothelial cells (ECs), cardiomyocytes, and smooth muscle cells [32,49,50,51,52,53].

At the cellular level, apoptosis consists of both the early exposure of membrane phosphatidylserine (PS) residues and the subsequent destruction of genomic DNA [54,55]. Externalization of membrane PS residues can occur first during cellular apoptosis [56,57]. Apoptotic membrane PS exposure occurs in neurons, vascular cells, and inflammatory microglia during reduced oxygen exposure [58,59,60,61,62], β-amyloid (Aβ) exposure [26,63], nitric oxide exposure [64,65,66,67,68], and during the administration of agents that induce the production of reactive oxygen species (ROS), such as 6-hydroxydopamine [69]. Membrane PS externalization also occurs on platelets and has been associated with clot formation in the vascular system [70]. Furthermore, membrane PS exposure can become a signal for the phagocytosis of cells [59,71,72]. The loss of membrane phospholipid asymmetry leads to the exposure of membrane PS residues on the cell surface and assists microglia to target cells for phagocytosis [4,52,73,74,75]. In conjunction with PS externalization, increased expression of the phosphatidylserine receptor (PSR) on microglia occurs to facilitate activation of these cells [76,77] since blockade of PSR function prevents the activation of microglia [74,78].

Usually following membrane PS exposure [79], the cleavage of genomic DNA into fragments occurs [35,61,80] as a later event during apoptotic injury [52,80,81,82]. There are a number of enzymes that degrade DNA. These include the acidic, cation independent endonuclease (DNase II), cyclophilins, and the 97 kDa magnesium - dependent endonuclease [83,84]. In addition, three separate endonuclease activities have been found in neurons that include a constitutive acidic cation-independent endonuclease, a constitutive calcium/magnesium-dependent endonuclease, and an inducible magnesium dependent endonuclease [85,86].

One of the inciting factors that can lead to apoptotic cell injury is oxidative stress. Oxidative stress plays a critical role in the pathology of numerous processes and disorders throughout the body that can include metabolic disorders [47,87,88,89,90,91,92,93,94,95], ocular disease [96], environmental influences such as with tobacco exposure [97,98], cognitive impairment [99,100,101,102], ischemic injury [103,105], epilepsy [106,107], nutrition [108], cardiopulmonary and hepatic disease [109,110,111], degenerative disorders and psychiatric disorders [112,113,114], infertility [115,116,117], excitotoxicity [118,119,120], and drug toxicity [121,122,123]. Early work with oxidative stress examined the rate of oxygen consumption in organisms and proposed that increased exposure to oxygen through a high metabolic rate could lead to a shortened life span [124]. Other work demonstrated that increased metabolic rates could be detrimental to animals in an elevated oxygen environment [125]. 

Recent studies have expanded these observations to show that ROS and mitochondrial DNA mutations have become associated with multiple processes to include cellular injury, aging mechanisms, and accumulated toxicity for an organism [126]. It is the release of ROS that leads to oxidative stress. ROS include superoxide free radicals, hydrogen peroxide, singlet oxygen, nitric oxide (NO), and peroxynitrite [34,83,127] that if expressed at increased concentrations can lead to cellular injury and demise through oxidative stress [59,128,129]. Most ROS occur at low levels and are scavenged by endogenous antioxidant systems that include superoxide dismutase (SOD), glutathione peroxidase, catalase, and small molecule substances such as vitamins C, D, E, and K [76,107,114,130,131]. Yet, one vitamin in particular, namely nicotinamide may be considered to stand-alone among antioxidants since nicotinamide influences multiple pathways tied to both cellular survival and cellular death.

**Figure 2:**
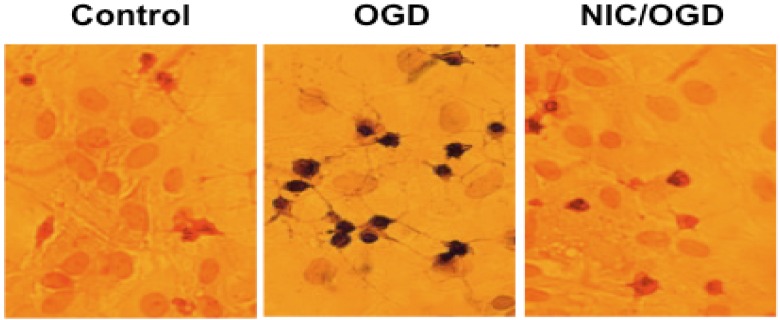
Nicotinamide prevents nuclear DNA fragmentation during oxidative stress with oxygen-glucose (OGD) deprivation**. **Representative images illustrate that nicotinamide (12.5 mM) with one hour pretreatment prior to OGD significantly blocked neuronal genomic DNA degradation assessed by terminal deoxynucleotidyl transferase nick end labeling (TUNEL) assay 24 hours after OGD.

In several scenarios, nicotinamide is a robust cytoprotectant that addresses both early membrane PS externalization and later genomic DNA degradation [3,4,34,76,132] during oxidative stress in a way that is different from other vitamin entities. Administration of nicotinamide during anoxia, oxygen-glucose deprivation, and free radical exposure can prevent exposure of membrane PS residues to block inflammatory cell activation [3,133,134,135] and inhibit later genomic DNA destruction [134,135,136] (Figure 2). In addition, nicotinamide prevents membrane PS exposure in vascular cells [4,134] that can reduce risk for cardiovascular disorders, since membrane PS residue externalization in vascular cells can lead to increased propensity for hypercoagulation [137] and cellular inflammation [138,139]. 

In several instances, nicotinamide also may reverse a previously sustained insult [4,132,133,134,135,140]. Post-treatment strategies with nicotinamide that can follow apoptotic injury in “real-time” show that cellular injury can be reversed. Nicotinamide can reverse an initial progression of membrane PS inversion and prevent PS exposure over a twenty-four hour period [4,132,135,141]. These results suggest that apoptosis prior to reaching genomic DNA degradation is dynamic and reversible in nature [4,132,135,141]. Yet, in not all cases may nicotinamide be effective to prevent subsequent DNA degradation [76]. During periods of acidosis-induced cellular toxicity [142], mitochondrial failure can ensue [143]. In addition, ROS can result in the disturbance of intracellular pH that leads to endonuclease activity and DNA injury during apoptosis [85,86,144]. In events that involve decreased pH, nicotinamide cannot prevent cellular injury during intracellular acidification [135]. For example, exposure to ROS leads to a biphasic response for pHi. Treatment with nicotinamide (12.5 mM) alone does not alter neuronal pHi. In addition, pretreatment with nicotinamide (12.5 mM), a neuroprotective concentration, 1 hour prior to ROS exposure does not significantly prevent the rapid acidification in neuronal cultures (pH 6.98 ± 0.06) during ROS exposure [135].

**Figure 3:**
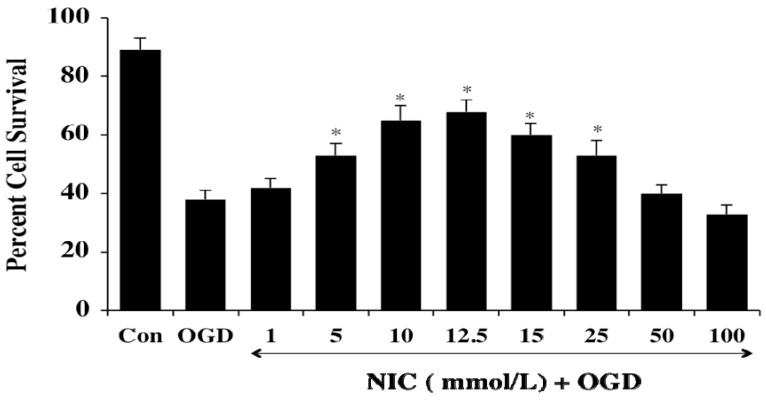
Nicotinamide leads to increased cell survival in a specific concentration range. Increasing concentrations of nicotinamide (1-100 mmol/L) were administered one hour prior to oxygen-glucose (OGD) deprivation to primary hippocampal neurons and cell injury was assessed 24 hours later with the trypan blue dye exclusion assay. Optimal cellular protection for nicotinamide occurs in the concentration range of 5.0 - 25.0 mmol/L.

Nicotinamide is considered to have protean endocrine effects [145,146], the ability to scavenge ROS, and offers cellular protection for both neuronal [140,147,148] and vascular cells [3,4,34,76]. In neuronal cell populations, nicotinamide protects against free radical injury [135], anoxia [132], excitotoxicity [149], homocysteine toxicity [150], ethanol-induced neuronal injury [151], and oxygen-glucose deprivation [140,152]. Nicotinamide prevents oxidant-induced apoptotic neuronal injury usually in a specific concentration range. Administration of nicotinamide in a range of 5.0-25.0 mmol/L significantly protect neurons during oxidative stress injuries (Figure 3). This concentration range is similar to other injury paradigms in both animal models [136] and in cell culture models [4,134,135]. In cortical neurons, nicotinamide antagonizes cell injury during ROS generating toxins such as tertiary butylhydroperoxide [153]. Nicotinamide also can protect both rod and cone photoreceptor cells against N-methyl-N-nitrosourea toxicity [136,154] as well as against glycation end products in all layers of the retina [155]. In animal studies, nicotinamide improves cognitive function, cell survival, and reduces edema following cortical trauma [156,157,158,159,160,161], limits axonal degeneration [162], reduces cerebral ischemia [163,164,165] sometimes more effectively in models that were absent of comorbidities [166], prevents spinal cord injury [167,168], and lessens disability in models of Parkinson’s disease in specific concentrations [169,170,171].

In addition to the observed neuroprotection with nicotinamide [140,147,148], the agent is also involved in the maintenance of vascular integrity [3,4,76]. For example, nicotinamide can protect the function of the blood brain barrier [156,157], influence arteriolar dilatation and blood flow [172], increase skin vascular permeability [173], potentially lead to decreased atherosclerotic plaque through inhibition of poly(ADP-ribose) polymerase [174], and promote platelet production through megakaryocyte maturation [175]. Nicotinamide also can maintain EC viability during ROS exposure [132,133,134,135,176]. Nicotinamide is believed to be responsible for the preservation of cerebral [177] and endocardial [178,179] ECs during models of oxidative stress [178,179]. However, recent reports suggest that pathways of nicotinamide also may have unclear vascular effects and may either prevent or contribute to atherosclerotic plaques over a three to six month progression [180]. It is possible that these events may occur during acidosis-induced cellular toxicity. During periods such as ischemia and oxidative stress, acidosis-induced cellular toxicity may ensue [142] and lead to subsequent mitochondrial failure [143]. Free radicals [86,144,181] can result in the disturbance of intracellular pH. In addition, modulation of intracellular pH is physiologically relevant for endonuclease activities during apoptosis [85,86,144]. As previously noted, nicotinamide cannot prevent cellular injury during intracellular acidification paradigms [135].

## 3. Nicotinamide and Inflammatory Cell Modulation

Closely tied to cellular survival and the ultimate disposal of non-functional cells is the activation of inflammatory cells [117,182]. As an example, when one considers disorders such as dementia [183] and inflammatory microglial cells of the brain, these cells can result in the phagocytic removal of both neurons and vascular cells [49,52,71]. During periods of inflammatory cell activation, microglia rely upon cytoprotective pathways [50,72] to proliferate and remove cells that are no longer functional [75,184]. Microglia can be beneficial in many ways to function as immune surveillance for toxic products [185], such as β-amyloid [186], block foreign microorganisms from entering the central nervous system and to allow for the repair of tissues composed of neuronal and vascular cells [50,187]. However, microglia have another side that may be detrimental to an organism. They can generate ROS [188,189], may worsen events with oxidative stress injury [190], and activate cytokines that in some circumstances may initially lead to cell proliferation [191], but later can lead to the demise of cells [192,193,194].

A number of cytoprotective agents rely upon the modulation of the immune system to control cellular survival. In particular, erythropoietin (EPO) is a prime example of a cytoprotective agent that is strongly associated with immune system pathways. Although EPO is approved by the Food and Drug Administration for the treatment of anemia and can have unwanted effects [127,195,196,197], it has recently been shown to significantly affect cell survival throughout the body [127,139,198,199], especially in regards to cellular proliferation [200,201,202,203]. EPO can reduce cytokine gene expression in endothelial cells exposed to tumor necrosis factor [204], prevent ulcer progression in cases of scleroderma [205], modulate inflammation during experimental autoimmune encephalomyelitis [206], reduce inflammation in murine arthritis models [207], and block primary microglial activation and proliferation during oxidative stress [25,78] to prevent phagocytosis of injured cells through pathways that involve cellular membrane PS exposure, protein kinase B (Akt) [49], and the regulation of caspases [78,208,209]. EPO can directly inhibit several pro-inflammatory cytokines, such as IL-6, tumor necrosis factor (TNF)-α, and monocyte chemoattractant protein 1 [199,210], and reduce leukocyte inflammation [211]. EPO also may foster the preservation of microglial cells for neuronal and vascular restructuring by preventing apoptotic injury in microglia [72,212].

EPO, although concentration dependent [78,138,208,213], can reduce cell injury during multiple events such as hyperoxia [214,215], hypoxia [78,138,216,217,218,219,220], parasitic infections [221,222,223], ROS exposure [64,208,224], ischemic/reperfusion insults [225,226,227,228,229], endotoxin shock [230,231], pulmonary disease [232,233,234], epileptic activity [235,236,237], elevated glucose exposure [238,239,240], excitotoxicity [224,241,242], mitochondrial failure [64,216,243], amyloid toxicity [25,244,245], cardiac and vascular injury [246,247,248,249,250,251,252], trauma [253,254,255,256], retinal disease [257], and renal failure [258,259,260].

Similar to agents such as EPO, nicotinamide can regulate cellular inflammation. Nicotinamide blocks pro-inflammatory cytokines, such as interleukin-1ß, interleukin-6, interleukin-8, tissue factor, and TNF-α [261,262,263,264] as well as transforming growth factor (TGF) β2, IL-1β, TNF-α, and macrophage chemotactic protein-1 in hepatic cells [265]. Nicotinamide affects major histocompatibility complexes [266], inhibits intracellular adhesion molecule expression [267], and modulates TNF in vascular cells [266] that may account for the ability of nicotinamide to reduce demyelination in models of multiple sclerosis [268]. Nicotinamide also may control inflammatory mechanisms that lead to arthritis, such as the inhibition of collagen II expression [269] as well as contact hypersensitivity reactions [270]. Yet, the role of nicotinamide during inflammation is not entirely clear, since some investigations that examined the ability of oral nicotinamide administration to reduce cytokine production following endotoxin challenge in healthy volunteers did not demonstrate a significant effect upon serum cytokine levels [271].

## 4. Nicotinamide, Metabolic Disease, and Energy Management

Nicotinamide may have an important role during cellular energy management and metabolic disorders such as diabetes mellitus (DM). DM affects both young and older individuals [15,16]. Almost 20 million individuals in the United States and more than 165 million individuals worldwide suffer from DM with increasing incidence [272]. By the year 2030, it is predicted that more than 360 million individuals will be afflicted with DM and its debilitating conditions. Type 2 DM represents at least 80 percent of all diabetics and is dramatically increasing in incidence as a result of changes in human behavior and increased body mass index [15,91]. Type 1 insulin-dependent DM is present in 5-10 percent of all diabetics and affects three million individuals in the United States alone, but is increasing at a rate of 4%, especially in adolescent minority groups [15,91]. Additional concerns are evident with the knowledge that a significant portion of the population has undiagnosed diabetes and impaired glucose tolerance, illustrating the need for improved early diagnosis [273]. 

Patients with DM can develop significant neurodegenerative [34,39,91], affective disorders [274], cognitive loss [275], and cardiovascular disease [91,276]. Interestingly, the development of insulin resistance and the complications of DM can be the result of cellular oxidative stress [15,91]. Hyperglycemia can lead to increased production of ROS in endothelial cells, liver cells, and pancreatic β-cells [15,16,91] and lead to apoptotic injury [55,239]. Recent clinical correlates support these experimental studies to show that elevated levels of ceruloplasmin are suggestive of increased ROS [15,16,91]. Furthermore, acute glucose swings in addition to chronic hyperglycemia can trigger oxidative stress mechanisms, illustrating the importance for therapeutic interventions during acute and sustained hyperglycemic episodes [15,91].

In regards to nicotinamide and its role during metabolic disorders, nicotinamide appears to have a close relationship with metabolic pathways that may lead to clinical cognitive effects [277]. Treatment with nicotinamide can maintain approximately normal fasting blood glucose with streptozotocin-induced DM in animal models [278,279]. Nicotinamide also can reduce peripheral nerve injury during elevated glucose [280], lead to the remission of type 1 DM in mice with acetyl-l-carnitine [281], and can inhibit oxidative stress pathways that lead to apoptosis [10,133,134,151,282]. Nicotinamide also affects levels of *O*-*N*-acetylglucosamin(*O*-GlcNAc)ylated proteins [283] and can significantly improve glucose utilization, prevent excessive lactate production and improve electrophysiologic capacity in ischemic animal models [284]. Oral nicotinamide administration (1,200 mg/m^2^/day) protects β-cell function and prevents clinical disease in islet-cell antibody-positive first-degree relatives of type-1 DM [285]. In addition, nicotinamide administration (25 mg/kg) in patients with recent onset type-1 DM combined with intensive insulin therapy for up to two years after diagnosis significantly reduced HbA_1c_ levels [286]. Potentially relevant to diabetic patients with renal failure, nicotinamide also has been shown to reduce intestinal absorption of phosphate and prevent the development of hyperphosphatemia and progressive renal dysfunction [287]. However, it is important to note that prolonged exposure to nicotinamide in some studies may lead to impaired β-cell function and reduction in cell growth [288,289]. Furthermore, nicotinamide also may inhibit P450 and hepatic metabolism [290] and play a role in the progression of other disorders such as Parkinson's disease [171].

Nicotinamide through NAD^+ ^has a critical physiological role in cellular metabolism and can be directly utilized by cells to synthesize NAD^+^ [4,34,76]. Nicotinamide also participates in energy metabolism through the tricarboxylic acid cycle by utilizing NAD^+^ in the mitochondrial respiratory electron transport chain for the production of ATP, DNA synthesis, and DNA repair [291,292,293]. Furthermore, nicotinamide can significantly increase NAD^+^ levels in vulnerable regions of the ischemic brain, suggesting that nicotinamide may offer cytoprotection of injured tissue through the maintenance of NAD^+^ levels [177]. During axonal degeneration, nicotinamide also may promote neuroprotection through NAD^+^-dependent mechanisms [162].

The preservation of cellular energy reserves is dependent upon the maintenance of mitochondrial integrity during DM [294]. ROS exposure can result in the opening of the mitochondrial membrane permeability transition pore [52,135,208,295], reduce mitochondrial NAD^+^ stores, and result in apoptotic cell injury [83]. Free fatty acids can lead to ROS release and contribute to mitochondrial DNA damage and impaired pancreatic β-cell function [296]. In patients with type 2 DM, skeletal muscle mitochondria have been described to be smaller than those in control subjects [297]. In addition, a decrease in the levels of mitochondrial proteins and mitochondrial DNA in adipocytes has been correlated with the development of type 2 DM [298]. Insulin resistance in the elderly also has been associated with elevation in fat accumulation and reduction in mitochondrial oxidative and phosphorylation activity [299]. An association also exists with insulin resistance and the impairment of intramyocellular fatty acid metabolism in young insulin-resistance offspring of parents with type 2 DM [300].

**Figure 4:**
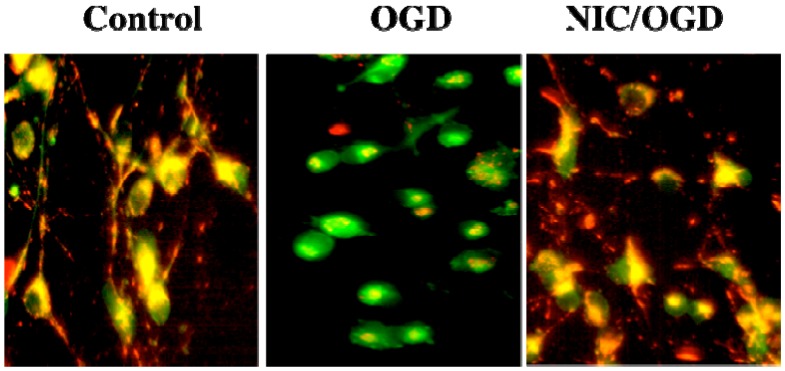
Nicotinamide prevents mitochondrial membrane depolarization during oxidative stress. Oxygen-glucose deprivation (OGD) produces a significant decrease in the red/green fluorescence intensity ratio of mitochondria using a cationic membrane potential indicator JC-1 within six hours when compared with untreated control cultures, demonstrating that OGD results in mitochondrial membrane depolarization. Pretreatment with nicotinamide one hour prior to OGD significantly increases the red/green fluorescence intensity of mitochondria, indicating that membrane potential is restored by nicotinamide.

Nicotinamide appears to function directly at the level of mitochondrial membrane pore formation [4,134,141] to prevent the release of cytochrome c [133] (Figure 4). Pretreatment of cells with either nicotinamide alone or in combination with the mitochondrial permeability transition pore inhibitor cyclosporin A prior to an injury paradigm can equally prevent mitochondrial membrane depolarization [301,302]. Nicotinamide can prevent the chemical induction of mitochondrial membrane depolarization during exposure to either *tert*-butylhydroperoxide or atractyloside [140]. There are additional pathways that nicotinamide may use to maintain cellular metabolic homeostasis through the maintenance of mitochondrial membrane potential [134,135]. Nicotinamide can phosphorylate Bad [133] to prevent mitochondrial membrane depolarization and subsequent cytochrome c release. In addition, nicotinamide may inhibit the assembly of the mitochondrial permeability transition pore complex similar to the action of cyclosporin A [303] as well as stabilize cellular energy metabolism since the maintenance of mitochondrial membrane potential is an ATP facilitated process [304].

## 5. Novel Intracellular Signaling for Nicotinamide

### 5.1. Forkhead transcription factors

Forkhead transcription factors of the “O” class (FoxOs) have recently been shown to mediate some of the biological effects of nicotinamide [305,306]. FoxO proteins either inhibit or activate target gene expression by binding bind to DNA through the forkhead domain that relies upon fourteen protein-DNA contacts [305,307,308,309,310]. According to X-ray crystallography [311] or nuclear magnetic resonance imaging [312], the forkhead domain is described as a "winged helix" as a result of a butterfly-like appearance. Members of this family that include FoxO1, FoxO3, FoxO4, and FoxO6 are found throughout the body and are expressed in tissues of the reproductive system of males and females, skeletal muscle, the cardiovascular system, lung, liver, pancreas, spleen, thymus, and the nervous system [117,309,310,313]. In addition, FoxOs govern a number of processes that involve cellular proliferation, degeneration, longevity, and neoplastic growth that may have associations with several novel pathways including *wingless* [26,55,77,184,314,315,316,317,318,319,320]. Other members of the forkhead family also rely upon *wingless* signaling that involve regulated as well as unchecked cellular growth [39,77,116,321,322].

Control of FoxO3a is a viable therapeutic target for agents such as metabotropic glutamate receptors [323], neurotrophins [324], cancer [117,309,325], and cytokines such as EPO [248] to increase cell survival. Recent work illustrates that FoxO3a may control early activation and subsequent apoptotic injury in microglia during Aβ exposure through caspase 3 [63]. Since Aβ exposure can facilitate the cellular trafficking of FoxO3a from the cytoplasm to the cell nucleus to potentially lead to apoptosis [63], one program in particular that may be vital for apoptotic injury appears to involve the activation of caspase 3. Aβ exposure leads to a rapid and significant increases in caspase 3 activity with six hours following Aβ administration, but that this induction of caspase 3 activity by Aβ requires FoxO3a, since loss of FoxO3a through gene silencing prevents the induction of caspase 3 activity by Aβ.

**Figure 5:**
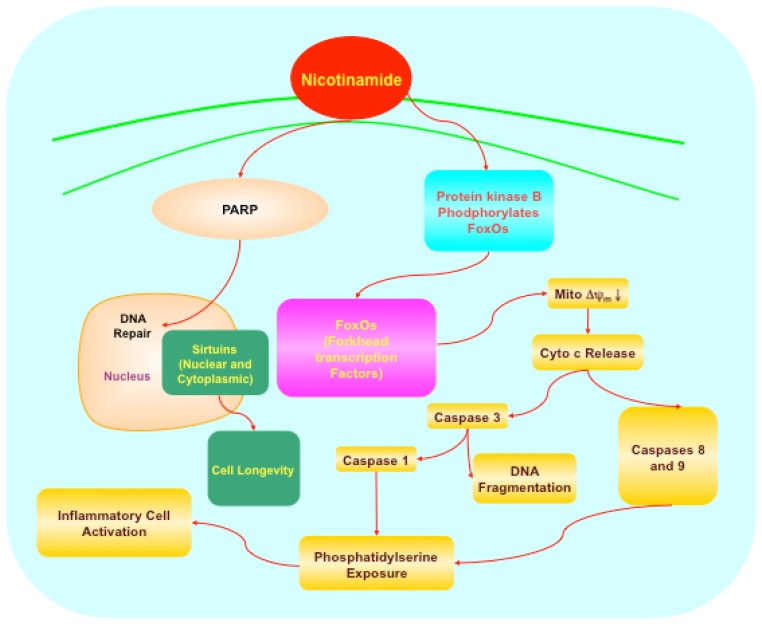
Nicotinamide relies upon novel cellular pathways to impact cell survival, longevity, and immune system function. Nicotinamide controls apoptotic early phosphatidylserine exposure, DNA repair and degradation, cell longevity, and immune cell activation through multiple pathways that involve modulation of sirtuin activity, protein kinase B (Akt), poly (ADP-ribose) polymerase (PARP), forkhead transcription factors, mitochondrial membrane potential (ΔΨ_m_), cytochrome c, (Cyto-c), and caspases 1,3, 8, and 9. These pathways can then regulate the onset of early apoptotic injury with phosphatidylserine exposure, late injury with nuclear DNA degradation, and inflammatory cell activation.

Nicotinamide has been shown to inhibit FoxO protein activity [140] and may be protective through two separate mechanisms of post-translational modification of FoxO3a [39,116,117,310,326] (Figure 5). Nicotinamide not only can maintain phosphorylation of FoxO3a and inhibit its activity to potentially block caspase 3 activity [140], but also can preserve the integrity of the FoxO3a protein to block FoxO3a proteolysis that can yield pro-apoptotic amino-terminal fragments [140]. During oxidative stress, an initial inhibitory phosphorylation of FoxO3a at the regulatory phosphorylation sites (Thr^32 ^and Ser^253^) occurs [140,196]. However, loss of phosphorylated FoxO3a expression appears to subsequently result over twelve hours, possibly by caspase degradation, which potentially can enhance the vulnerability of neurons to apoptotic injury [140]. The loss of both FoxO3a phosphorylation and the integrity of this transcription factor may then lead to apoptosis. FoxO3a proteolysis occurs during cell injury yielding an amino-terminal (Nt) fragment that can become biologically active and lead to cellular injury [327]. Nicotinamide, through the phosphorylation of FoxO3a at regulatory sites that possess high affinity for protein kinase B (Akt) can prevent apoptotic cell injury [140]. In addition, modulation of caspase 3 activity by nicotinamide appears to be tied to a unique regulatory mechanism that blocks the proteolytic degradation of phosphorylated FoxO3a by caspase 3. Since FoxO3a has been shown to be a substrate for caspase 3-like proteases at the consensus sequence DELD^304^A [327], blockade of caspase 3 activity prevents the destruction of phosphorylated FoxO3a during oxidative stress [140], suggesting that nicotinamide maintains a regulatory loop through the modulation of caspase 3 and the preservation of phosphorylated FoxO3a integrity.

FoxO proteins also have been associated with cell longevity and aging as shown by early studies linking DAF-16 in *Caenorhabditis elegans* to increased longevity as well as the association with sirtuins [117,306,328,329,330]. Yet, the relationship among nicotinamide, FoxO transcription factors, and proteins that increased cellular lifespan is not entirely clear. For example, the sirtuin Sirt1 is a NAD^+^-dependent deacetylase and the mammalian ortholog of the silent information regulator 2 (Sir2) protein associated with increased lifespan in yeast. Some studies suggest that stimulation of Sirt1 during starvation is dependent upon FoxO3a activity as well as p53 [331]. In addition, during exercise, an up-regulation of FoxO3a and Sirt1 activity is observed in the heart [332], suggesting that physical activity may be beneficial for the cardiovascular system through FoxO proteins. Yet, other work has shown that Sirt1 may repress the activity of FoxO1, FoxO3a, and FoxO4, suggesting that cellular longevity may benefit from reduction in FoxO protein generated apoptosis [333]. 

In regards to nicotinamide, cellular protection and longevity, it appears that a reduction in nicotinamide levels during nicotinamidase expression supports increased cellular survival and longevity [334,335] (Figure 5). Nicotinamide can block cellular Sir2 by intercepting an ADP-ribosyl-enzyme-acetyl peptide intermediate with the regeneration of NAD^+^ (transglycosidation) [336]. Nicotinamidase expression which reduces nicotinamide concentrations prevents both apoptotic late DNA degradation and early PS exposure that may serve to modulate inflammatory cell activation. In addition, inhibition of sirtuin (Sirt1) activity either by pharmacological methods or siRNA gene silencing is detrimental to cell survival during oxidative stress and blocks nicotinamidase protection, further supporting that Sirt1 activity may be necessary for nicotinamidase protection during oxidative stress. Furthermore, nicotinamide offers gene regulation [337] and cellular protection in millimole concentrations against oxidative stress. Physiological concentrations of nicotinamide noncompetitively inhibit Sir2, suggesting that nicotinamide is a physiologically relevant regulator of Sir2 enzymes [338]. As a result, in relation to cell longevity, it is the lower concentrations of nicotinamide that can function as an inhibitor of sirtuins that are necessary for the promotion of increased lifespan and cellular survival [132,133,134,140,334,335,339], at least in yeast and metazoans [76,340,341]. Interestingly, it has been postulated that sirtuins also may prevent nicotinamide from assisting with DNA repair by altering the accessibility of DNA damaged sites for repair enzymes [342].

### 5.2. Protein kinase B (Akt), Bad, caspases, and mitogen-activated protein kinases

Post-translational modulation of FoxO proteins occurs through phosphorylation, acetylation, and ubiquitylation [306,326]. The serine-threonine kinase protein kinase B (Akt) is a principal pathway of phosphorylation of FoxOs that can block activity of these proteins [305,343]. Activation of Akt is usually “pro-survival” and cytoprotective, such as during cell proliferation [344], hyperglycemia [345], ischemia/stress [346,347], hypoxia [216], β-amyloid (Aβ) toxicity [25], cardiomyopathy [348], cellular aging [349], neurodegeneration [350,351], and oxidative stress [49,52,74]. Akt can prevent cellular apoptosis through the phosphorylation of FoxO proteins [117,309] and maintain FoxO transcription factors in the cytoplasm by association with 14-3-3 proteins and prevent the transcription of pro-apoptotic target genes [199,248]. 

Cytoprotection through Akt also can involve control of apoptotic inflammatory cell activation [52,74,208], maintenance of mitochondrial membrane potential (ΔΨ_m _), and prevention of cytochrome c release [64,78,208]. Akt can prevent early apoptotic membrane PS exposure on injured cells and block the activation of microglia during oxidative stress [52,71,74]. Nicotinamide uses mechanisms that involve Akt to regulate microglial activation and proliferation [133,140] by blocking membrane PS exposure on cells and possibly preventing the shedding of membrane PS residues that is known to occur during apoptosis [352] (Figure 5). 

Akt also regulates pathways that involve Bad, a pro-apoptotic Bcl-2 family member that becomes active through phosphorylation on its serine residues [83]. Phosphorylation of Bad by Akt leads to the binding of Bad with the cytosolic protein 14-3-3 to release Bcl-x_L _and allows Akt to block apoptosis [353]. Bcl-2 and Bcl-x_L_ block Bax translocation to the mitochondria, maintain mitochondrial membrane potential, and prevent the release of cytochrome c from the mitochondria [208,354]. Nicotinamide can promote the phosphorylation of Bad during oxidative stress [133]. This phosphorylation of Bad by nicotinamide can be blocked by lack of Akt activity, suggesting that nicotinamide phosphorylates Bad through an Akt mediated pathway [343]. In addition, Akt may promote cell survival through the inhibition of apoptotic p53 transcriptional activity [355] that may be regulated by nicotinamide. Nicotinamide also has been shown to either directly limit the expression of p53 [153] or prevent an NAD-dependent p53 deacetylation induced by Sir2α [356].

Since Akt can prevent caspase activity [78,208,216], it is conceivable to assume that nicotinamide also may regulate caspase activity. Caspases are a family of cysteine proteases that are synthesized as inactive zymogens which are proteolytically cleaved into subunits during apoptosis [76,357,358]. The apoptotic-associated caspases include initiator caspases, such as caspase 2, 8, 9, and 10, that activate downstream effector caspases, resulting in an amplification of cascade activity. The initiator caspases consist of long N-terminal prodomains that contain caspase recruitment domains (CARDs) in caspase 2 and caspase 9 or death effector domains (DEDs) in caspase 8 and caspase 10. The effector caspases consist of caspase 3, 6, and 7 that function to directly cleave crucial cellular protein substrates to result in cell destruction [55,83,142,357]. Caspase 8 is as an upstream initiator of executioner caspases, such as caspase 3, and also leads to the mitochondrial release of cytochrome c [359,360]. Following caspase 8 and caspase 9 activation, caspase 3 directly leads to genomic DNA degradation [52,74,78].

Caspases 1 and 3 mediate genomic DNA cleavage and cellular membrane PS exposure [64,208,361]. These caspases [4,134,135,141], in addition to caspase 8 and 9, are also tied to the direct activation and proliferation of microglia [52,74,78]. Caspase 1 is believed to be principally responsible for the externalization of membrane PS residues in several cell systems that can subsequently activate microglial phagocytosis [80,362]. Furthermore, caspase 9 is activated through a process that involves the cytochrome c -apoptotic protease-activating factor-1 (Apaf-1) complex [79,363]. In regards to membrane PS exposure, nicotinamide prevents PS externalization primarily through the inhibition of caspase 1 -like activity [133] (Figure 5). Nicotinamide also prevents genomic DNA cleavage as well as early membrane PS exposure through caspase 8 and caspase 9 - like activities [4,133,134,135,140]. The precise pathways that are necessary for nicotinamide to modulate caspase pathways remain under investigation. Although some "anti-apoptotic" proteins, such as EPO [199,208] modulate both Apaf-1 expression and cytochrome c release, protection through nicotinamide remains independent from Apaf-1 [140]. However, nicotinamide can significantly prevent cell injury by inhibiting caspase 9 - like activity directly [140].

Nicotinamide also relies upon the stress activated family of mitogen-activated protein kinases (MAPK) that includes the p38 kinases (MAPK^p38^) and the c-Jun N-terminal kinases (MAPK^JNK^). The family of MAPKs consists of the subgroups that include ERK1 (MAPK^ERK1/p44^), ERK2 (MAPK^ERK2/p42^), the JNKs (MAPK^JNK^), and p38 MAPKs (MAPK^p38^). Although significant activation of MAPK^p38^ and MAPK^JNK^ is present in cells during oxidative stress [4,132,135,141] and c-Jun leads to apoptosis through transcription activation of some pro-apoptotic genes [364], nicotinamide does not appear to alter the activity of either MAPK^p38^ or MAPK^JNK^ [133]. These results suggest that nicotinamide cytoprotection does not require the MAPK^p38^ and MAPK^JNK^ pathways [4,132,135,141].

### 5.3. Poly (ADP-ribose) polymerase (PARP)

Nicotinamide has an intimate relationship with poly (ADP-ribose) polymerase (PARP) that also been recently associated with both vascular and neurodegenerative disorders [76,174,365] (Figure 5). PARP is a nuclear protein that binds to DNA strand breaks and cleaves NAD^+^ into nicotinamide and ADP-ribose. PARP catalyses the synthesis of poly (ADP-ribose) from its substrate NAD^+^, which stimulates the process of DNA repair [366]. Nicotinamide concentrations of at least 1 mM have been shown to provide sufficient stores of NAD^+^ for PARP activation [367]. Nicotinamide can prevent PARP degradation and allow for DNA repair through the direct inhibition of caspase 3 [133,134,135]. In contrast, elevated concentrations of nicotinamide can lead to PARP degradation and apoptotic injury [368]. 

However, other work illustrates that a reduction in PARP activity may enhance cell survival, such as during injury paradigms with photoreceptor cells [369], during homocysteine toxicity [370], during cerebral ischemia [371], or during free radical injury [372,373]. Prevention of NAD^+ ^depletion during enhanced PARP activity also has been demonstrated to prevent cellular lysis during oxidative stress [374]. In human blood lymphocytes during oxidative stress, nicotinamide may block necrotic death through pathways that limit excessive PARP activity that can consume essential NAD^+^ stores [375]. During diabetic neuropathy, nicotinamide reduces PARP activity to partially restore vital NAD^+^ and ATP [376].

Inhibition of PARP activity by nicotinamide also may be critical for disorders such as Alzheimer's disease. The National Institute on Aging estimates that almost five million people in the United States have Alzheimer’s disease and worldwide more than twenty-four million people suffer from Alzheimer’s disease, pre-senile dementia, and other disorders of cognitive loss. In Alzheimer's disease [99], Aβ is toxic to cells [25,26,121] and is associated with the phosphorylation of the forkhead transcription factors that can be blocked by scavengers of oxidative stress [377]. A prior pilot study has suggested that administration of nicotinamide adenine dinucleotide (NADH) in patients with Alzheimer’s may show improvement in their cognitive function [378]. More recently, dietary niacin intake examined in a series of patients aged 65 and older has been implicated as a protectant against the development or progression of Alzheimer's disease [379]. Interestingly, it has been shown that in patients with Alzheimer's disease, both PARP and poly(ADP-ribose) is present in the frontal and temporal cortex more frequently than in controls, suggesting that increased levels of functional PARP enzyme are present to potentially lead to the depletion of NAD^+^ stores [380]. In addition, Aβ toxicity may require increased PARP activity [381].

## 6. Conclusions

Therapeutic innovation relies heavily upon the understanding and emerging knowledge of the cellular pathways that govern disease. Yet, no therapeutic modality can be used with clear focus and caution to gain the greatest clinical efficacy with the least amount of unwanted detrimental effects. As a result, nicotinamide clearly comes under such constraints. As an agent that offers broad cytoprotective effects that may be applicable to multiple disorders of the cardiovascular, nervous, immune, and metabolic pathways, nicotinamide also has complex biological roles. For example, nicotinamide pathways that rely upon FoxO modulation may not consistently lead to controlled enhanced cell survival, but rather with unchecked cellular proliferation that leads to cancer. Furthermore, nicotinamide offers a fine line with concentration administration with lower concentrations of nicotinamide possibly negating sirtuin activity and decreasing lifespan in organisms. In addition, protection against PARP or preservation of its activity by nicotinamide also may sometimes lead to unwanted outcomes. PARP activation can deplete NAD^+^, lower ATP production, and precipitate cell death. Under other conditions, nicotinamide has been described as an agent that limits cell growth and promotes cell injury. Nicotinamide in the presence of transforming growth factor ß-1 can block hepatic cell proliferation and lead to apoptosis with caspase 3 activation [265]. During moderate temperature hyperthermia or carbogen breathing, nicotinamide also can result in enhanced solid tumor radiosensitivity and assist with tumor load reduction [382]. With these considerations for nicotinamide, it is clear that this agent holds great potential for multiple disease entities, but the development of new therapeutic strategies with nicotinamide rests heavily upon the elucidation of the intimate relationship nicotinamide holds with novel pathways that include forkhead transcription factors, sirtuins, Akt, caspases, MAPK, and PARP.
